# Supplementary material for: Single Cell Transcriptomic and Chromatin Profiles Suggest Layer Vb Is the Only Layer With Shared Excitatory Cell Types in the Medial and Lateral Entorhinal Cortex
Source: Front Neural Circuits. 2022 Jan 26;15:806154. doi: 10.3389/fncir.2021.806154 (PMC8826650; doi:10.3389/fncir.2021.806154)
Supplement: Supplementary file 14 [file Data_Sheet_1.ZIP › Exc_vs_Inh_Motifs/homerResults.html]

Exc\_vs\_Inh\_Motifs// - Homer de novo Motif Results


# Homer *de novo* Motif Results (Exc\_vs\_Inh\_Motifs//)

Known Motif Enrichment Results  
Gene Ontology Enrichment Results  
If Homer is having trouble matching a motif to a known motif, try copy/pasting the matrix file into
STAMP  
More information on motif finding results: HOMER
| Description of Results
| Tips
  
Total target sequences = 42187  
Total background sequences = 10872  
\* - possible false positive  

|  |  |  |  |  |  |  |  |  |
| --- | --- | --- | --- | --- | --- | --- | --- | --- |
| Rank | Motif | P-value | log P-pvalue | % of Targets | % of Background | STD(Bg STD) | Best Match/Details | Motif File |
| 1 | T G A C T C A G G A T C C G T A C A T G C T A G C G A T C T A G A G T C C A T G | 1e-3486 | -8.029e+03 | 41.61% | 15.75% | 232.0bp (254.8bp) | E2A(bHLH),near\_PU.1/Bcell-PU.1-ChIP-Seq(GSE21512)/Homer(0.982) More Information \| Similar Motifs Found | motif file (matrix) |
| 2 | T G A C G T A C C G T A A C G T T G C A C G A T A C T G A C T G G A C T G T C A | 1e-1307 | -3.010e+03 | 18.40% | 6.99% | 218.3bp (267.2bp) | Atoh1/MA0461.2/Jaspar(0.953) More Information \| Similar Motifs Found | motif file (matrix) |
| 3 | C A T G A G C T T C A G A C T G A T C G G A T C C T A G C A T G | 1e-903 | -2.080e+03 | 33.31% | 19.93% | 242.4bp (245.3bp) | Egr2(Zf)/Thymocytes-Egr2-ChIP-Seq(GSE34254)/Homer(0.932) More Information \| Similar Motifs Found | motif file (matrix) |
| 4 | C G T A A G T C C G T A G A T C T A G C C A G T A C G T A T C G | 1e-389 | -8.971e+02 | 58.27% | 48.01% | 251.0bp (260.2bp) | TBX21/MA0690.1/Jaspar(0.692) More Information \| Similar Motifs Found | motif file (matrix) |
| 5 | C T G A A T G C T G C A A G C T C G T A A C G T C T G A A C G T | 1e-350 | -8.077e+02 | 59.24% | 49.51% | 250.6bp (289.0bp) | PB0163.1\_Six6\_2/Jaspar(0.839) More Information \| Similar Motifs Found | motif file (matrix) |
| 6 | A C G T C T G A C G T A A G T C C G T A A G C T A C G T C G A T | 1e-314 | -7.248e+02 | 25.29% | 17.88% | 246.5bp (279.4bp) | Arid5a/MA0602.1/Jaspar(0.750) More Information \| Similar Motifs Found | motif file (matrix) |
| 7 | C A G T A C T G T A G C A G T C G T C A T C G A T A G C C G T A | 1e-310 | -7.150e+02 | 37.47% | 28.94% | 243.4bp (233.1bp) | NFIA/MA0670.1/Jaspar(0.909) More Information \| Similar Motifs Found | motif file (matrix) |
| 8 | C A G T C G A T C G T A C G T A C T G A T C G A G C T A T C G A | 1e-291 | -6.708e+02 | 57.50% | 48.62% | 246.4bp (269.4bp) | ZNF384/MA1125.1/Jaspar(0.820) More Information \| Similar Motifs Found | motif file (matrix) |
| 9 | C T G A C A G T C T A G A G C T C G T A G T A C G A T C G C T A G T A C G T C A | 1e-241 | -5.568e+02 | 65.49% | 57.57% | 248.4bp (263.6bp) | DMRT3/MA0610.1/Jaspar(0.741) More Information \| Similar Motifs Found | motif file (matrix) |
| 10 | G C A T G C T A G C A T C G A T G C T A G C T A G C T A C G A T C G A T G C A T G C T A C G T A | 1e-241 | -5.554e+02 | 33.89% | 26.57% | 241.6bp (276.9bp) | PB0068.1\_Sox1\_1/Jaspar(0.673) More Information \| Similar Motifs Found | motif file (matrix) |
| 11 | C G T A A C G T G T A C A G T C A T G C G T C A A G T C C T G A | 1e-226 | -5.218e+02 | 50.46% | 42.67% | 254.7bp (248.3bp) | ZNF75D/MA1601.1/Jaspar(0.721) More Information \| Similar Motifs Found | motif file (matrix) |
| 12 | C T G A C G A T A G T C G C A T T C G A G A C T G A T C G A C T G C T A G A C T A G T C G A C T | 1e-186 | -4.290e+02 | 59.62% | 52.57% | 260.0bp (261.6bp) | GATA3(Zf),DR4/iTreg-Gata3-ChIP-Seq(GSE20898)/Homer(0.706) More Information \| Similar Motifs Found | motif file (matrix) |
| 13 | A G T C A C G T C G T A A G T C C G T A A C T G A C T G A C G T C G T A C G T A A G T C C G T A | 1e-175 | -4.035e+02 | 40.32% | 33.73% | 254.6bp (255.9bp) | PH0083.1\_Irx3\_1/Jaspar(0.668) More Information \| Similar Motifs Found | motif file (matrix) |
| 14 | T A G C G A T C G C A T A T G C T C A G G C T A C G T A A G T C G A C T T G A C | 1e-174 | -4.023e+02 | 8.77% | 5.41% | 258.0bp (258.1bp) | ZNF135/MA1587.1/Jaspar(0.730) More Information \| Similar Motifs Found | motif file (matrix) |
| 15 | T C A G A G T C A C G T C T A G A C T G A C T G C G T A C G A T C G A T C G T A C G T A C G T A | 1e-166 | -3.844e+02 | 1.48% | 0.39% | 259.9bp (341.8bp) | PH0124.1\_Obox5\_1/Jaspar(0.735) More Information \| Similar Motifs Found | motif file (matrix) |
| 16 | C T A G C G A T A C T G C G A T A C T G G C A T C T G A C A T G T A C G C G A T C T G A C A T G | 1e-165 | -3.802e+02 | 0.52% | 0.05% | 269.6bp (32.8bp) | PB0155.1\_Osr2\_2/Jaspar(0.628) More Information \| Similar Motifs Found | motif file (matrix) |
| 17 | C G T A A G T C C G T A C G T A A C G T A G T C A C G T A C T G A C G T A C G T | 1e-159 | -3.671e+02 | 7.39% | 4.45% | 242.2bp (267.7bp) | NeuroG2(bHLH)/Fibroblast-NeuroG2-ChIP-Seq(GSE75910)/Homer(0.771) More Information \| Similar Motifs Found | motif file (matrix) |
| 18 | C T G A T C A G G T C A T C G A C A G T T A C G T C A G T A C G C G A T T C A G C T G A C T A G | 1e-153 | -3.527e+02 | 52.18% | 45.76% | 250.2bp (241.9bp) | Nfe2l2/MA0150.2/Jaspar(0.657) More Information \| Similar Motifs Found | motif file (matrix) |
| 19 | C G T A A C T G A C G T A C T G G T A C A C G T C T A G A C T G A C T G C G T A | 1e-129 | -2.993e+02 | 2.71% | 1.21% | 262.1bp (247.5bp) | Stat5b/MA1625.1/Jaspar(0.686) More Information \| Similar Motifs Found | motif file (matrix) |
| 20 | A C G T C A T G A C T G C G T A T G C A A C T G C G T A A C T G A G T C C G T A A C T G A C G T | 1e-118 | -2.733e+02 | 0.33% | 0.02% | 212.8bp (201.8bp) | ZNF189(Zf)/HEK293-ZNF189.GFP-ChIP-Seq(GSE58341)/Homer(0.684) More Information \| Similar Motifs Found | motif file (matrix) |
| 21 | A C G T A C G T A C G T A C G T A C T G A C G T A C G T C G T A A C G T A C T G | 1e-117 | -2.711e+02 | 63.15% | 57.63% | 246.6bp (267.5bp) | PB0123.1\_Foxl1\_2/Jaspar(0.816) More Information \| Similar Motifs Found | motif file (matrix) |
| 22 | A G T C A C G T A G T C A G T C C G T A C T G A G T A C C G T A A C G T C A T G G C A T C G T A | 1e-117 | -2.705e+02 | 0.33% | 0.02% | 212.7bp (105.2bp) | PH0086.1\_Irx5/Jaspar(0.689) More Information \| Similar Motifs Found | motif file (matrix) |
| 23 | A C T G A C G T C G T A A G T C C G T A C G T A A G T C A G T C C G T A A G T C | 1e-113 | -2.623e+02 | 92.52% | 89.27% | 255.0bp (252.5bp) | RUNX1(Runt)/Jurkat-RUNX1-ChIP-Seq(GSE29180)/Homer(0.681) More Information \| Similar Motifs Found | motif file (matrix) |
| 24 | A G C T A C T G A C G T A C G T T G A C C G T A A C G T C G T A A C T G A T G C C G T A A T C G | 1e-109 | -2.513e+02 | 0.40% | 0.04% | 238.5bp (115.1bp) | Foxf1(Forkhead)/Lung-Foxf1-ChIP-Seq(GSE77951)/Homer(0.606) More Information \| Similar Motifs Found | motif file (matrix) |
| 25 | A G T C A C G T C G T A A C T G A T G C C G T A A G C T A T C G | 1e-81 | -1.887e+02 | 5.46% | 3.60% | 255.2bp (236.1bp) | PB0181.1\_Spdef\_2/Jaspar(0.740) More Information \| Similar Motifs Found | motif file (matrix) |
| 26 | C T G A C G T A T G C A C A G T A C T G C T A G C G T A C G T A A C T G A T C G A C G T A C T G | 1e-79 | -1.824e+02 | 0.47% | 0.09% | 211.5bp (286.2bp) | TEAD2/MA1121.1/Jaspar(0.771) More Information \| Similar Motifs Found | motif file (matrix) |
| 27 | A C T G A C G T C G T A A T C G A G T C A T G C A G T C A C G T A C T G A C T G | 1e-54 | -1.252e+02 | 0.90% | 0.36% | 241.3bp (335.6bp) | PB0051.1\_Osr2\_1/Jaspar(0.657) More Information \| Similar Motifs Found | motif file (matrix) |
| 28 | C G T A A C G T C G T A C G T A A C T G A C G T A C T G A C T G C G T A C G A T | 1e-47 | -1.093e+02 | 0.42% | 0.11% | 211.4bp (145.4bp) | Nkx3-2/MA0122.3/Jaspar(0.811) More Information \| Similar Motifs Found | motif file (matrix) |
| 29 | A C T G A C G T A G T C A C G T A C G T A G T C A C G T A C T G A C T G A G T C | 1e-41 | -9.483e+01 | 0.25% | 0.05% | 239.7bp (162.4bp) | Hand1::Tcf3/MA0092.1/Jaspar(0.670) More Information \| Similar Motifs Found | motif file (matrix) |
| 30 | C G T A C G T A C G T A A C T G A C T G A C G T C G T A C G T A A C G T A G T C A C G T C G T A | 1e-34 | -7.834e+01 | 0.38% | 0.12% | 240.0bp (177.2bp) | Nur77(NR)/K562-NR4A1-ChIP-Seq(GSE31363)/Homer(0.684) More Information \| Similar Motifs Found | motif file (matrix) |
| 31 | G A T C G C T A G C A T G C T A G C A T G C T A G A T C G T A C G T A C G T C A T C G A C G A T | 1e-32 | -7.521e+01 | 0.36% | 0.12% | 248.9bp (56.3bp) | PB0163.1\_Six6\_2/Jaspar(0.588) More Information \| Similar Motifs Found | motif file (matrix) |
| 32 | C G T A A G T C C G T A C G T A A G T C C G T A A C G T C G T A A C G T A G T C C G T A A G T C | 1e-29 | -6.855e+01 | 0.32% | 0.11% | 238.3bp (64.3bp) | NEUROG2/MA0669.1/Jaspar(0.629) More Information \| Similar Motifs Found | motif file (matrix) |
| 33 | C G T A C G T A A C T G C G T A A G T C A G T C A G T C A C G T A C T G A C G T | 1e-28 | -6.626e+01 | 0.23% | 0.06% | 252.5bp (116.5bp) | LRF(Zf)/Erythroblasts-ZBTB7A-ChIP-Seq(GSE74977)/Homer(0.735) More Information \| Similar Motifs Found | motif file (matrix) |
| 34 | A G T C A C T G A C G T A C G T A C T G A C T G A C G T A G T C | 1e-24 | -5.654e+01 | 0.21% | 0.06% | 211.2bp (184.0bp) | ZBTB7C/MA0695.1/Jaspar(0.712) More Information \| Similar Motifs Found | motif file (matrix) |
